# Optimization of a Method to Prepare Liposomes Containing HER2/Neu- Derived Peptide as a Vaccine Delivery System for Breast Cancer 

**Published:** 2014

**Authors:** Sheyda Shariat, Ali Badiee, Mahmoud Reza Jaafari, Seyed Alireza Mortazavi

**Affiliations:** a *Department of Pharmaceutics, School of Pharmacy, Shahid Beheshti University of Medical Sciences, Tehran, Iran.*; b *Nanotechnology Research Center, School of Pharmacy, Mashhad University of Medical Sciences, Mashhad, Iran. *; c *Biotechnology Research Center, Nanotechnology Research Center, School of Pharmacy, Mashhad University of Medical Sciences, Mashhad, Iran. *

**Keywords:** P5 peptide, HER2/neu protein, Tumor vaccine, Encapsulation, Liposome

## Abstract

The purpose of this study was to optimize a method for the encapsulation of P5 peptide, a new designed peptide containing MHC class I epitopes from rat HER2/neu protein, into liposomes as an approach for breast cancer vaccine formulation. The efficiency of liposomal encapsulation of peptides is generally low and development of an optimized method to increase encapsulation efficiency is a big challenge. In this study, P5 peptide was encapsulated into liposomes using the following three different methods based on film-hydration procedure. In method A, the lipid film containing P5 was hydrated using buffer and then extruded to 100 nm using polycarbonate filter. In method B all the steps were the same as method A, except that the lipid film was hydrated in buffer containing 10% (v/v) of DMSO and P5 peptide. In method C, P5 peptide was added to preformed liposomes (40 mM) in the presence of ethanol (30% v/v) and incubated at 25 ºC for 1h. The highest peptide encapsulation efficiency was achieved using method C (44%). The presence of P5 peptide in purified liposomes was also confirmed using SDS- PAGE analysis. Investigation on the effects of procedure parameters of method C on encapsulation efficiency demonstrated that method is an optimized procedure for encapsulating P5 peptide. Maximal recovery from liposomes for the accurate quantification of peptide was discovered using acidified isopropanol at 1:2 of sample to solvent ratio (v/v). In conclusion, the optimal methods of encapsulation and peptide content determination in liposomes can accelerate the development of liposomal vaccine formulations**. **

## Introduction

In recent years, liposomes have been widely used as beneficial carriers of protein or peptide antigens for vaccines (1). Among different strategies in using these lipid carriers to induce immune responses in various ailments, attempts to encapsulate peptides derived from tumor-associated antigens (TAA) in liposomes to achieve effective anti-tumor vaccines are considerable. This interest can be attributed to this finding that encapsulation of peptide antigens in liposomes protects them from degradation and enhances their immunogenicity (2). The ability of liposomes to increase immune responses has been attributed primarily to an increased antigen uptake by antigen presenting cells (APCs) and consequently enhanced antigen presentation to T cells (3). Moreover, several advantages that TAA offer over other antitumor vaccination strategies including cost-effective production ease of manufacturing, chemical stability ease of quality control and lack of toxic or infectious materials in vaccine formulation (4).

Typical methods such as lipid film hydration or hand shaking, sonication, freeze-dried rehydration, reverse phase evaporation, and detergent depletion can be used to encapsulate peptide antigens in liposomes (5).

Efficiency of liposomal encapsulation of peptides is generally low and common methods used for encapsulating peptides result in very low encapsulation efficiencies. For example, loss of peptides on filters often occurs in using film extrusion method. Aggregation of peptides might be happened due to application of energy when sonication is used (6). Optimization of an appropriate method to increase encapsulation efficiency would be a challenge for researchers in this field. To develop an optimal method, understanding of the fundamental mechanisms of encapsulation and identification of parameters that influence entrapment efficiency should be carefully considered. Formulation and production process parameters such as lipid type and concentration, peptide concentration, buffer’s pH and ionic strength, surface charge (7-9), vesicle preparation method (10,11), number of freeze-thawing cycles and time of each cycle (12,13) affect the liposomal encapsulation yield of peptides or proteins.

The dose of the peptide antigen can significantly influence the type and magnitude of immune responses. Therefore, the development of methods that determine peptide content in liposomes accurately is the next challenge in vaccine formulation. Encapsulated peptide can be extracted from liposomes by various extraction media including Triton X-100 (14, 15), sodium dodecyl sulfate (16), methanol and ethanol (17), acidified isopropanol (18), n-butanol and sodium bicarbonate (19). In selecting the suitable medium for extracting a particular peptide, the actual recovery value and its reproducibility must be considered. Once the encapsulated peptide is separated from the lipids, it can be quantified by several accurate methods including RP-HPLC.

A hydrophobic peptide containing MHC class I restricted multi-epitopes from rat HER2/neu protein, called P5 hereafter, was used as a peptide antigen in this study. In our previous study, P5 was designed by *in-silico *analysis and the effectiveness of it was evaluated by injection to the BALB/c mice (20). The results showed P5 was effective in inducing CTL response. It was hypothesized that encapsulating P5 in lipid carriers may enhance CTL immune response more than peptide alone. Although encapsulating peptide in LPD (liposome-polycation-DNA) nanoparticles included DOTAP and CpG as immune-stimulatory components by Jalali et al. confirmed this hypothesis (20), however based on LPD nanoparticles’ complexity and some cellular toxicity (21) highlighted the use of liposomes as simpler and safer carriers for vaccine delivery. Liposomes are FDA approved adjuvants, the results of clinical trials have shown that liposomes are safe and well-tolerated (22). 

In the present study, we optimized film-hydration method for encapsulating P5 peptide in liposomes efficiently. The effects of various preparation parameters such as lipid, ethanol concentration, incubation time and temperature on encapsulation efficiency were investigated in order to obtain high encapsulation yield. We also selected an effective extraction medium for separating peptide P5 from lipids and to determine the peptide content in liposomes accurately. Development of an optimized method to encapsulate and analyze peptides in liposomes can accelerate utilizing liposomal vaccine formulations. 

## Experimental

*Materials*

Peptide P5 (ELAAWCRWGFLLALLPPGIAG, Purity > 95%) was synthesized by Peptron Co. (Daejeon, South Korea). Dimiristoylphosphatidylcholine (DMPC) and Dimiristoylphosphoglycerol (DMPG) were purchased from Avanti Polar Lipid (Alabaster, USA). Cholesterol, L- ascorbic acid, ammonium molybdate, Tris and Tricine were from Sigma-Aldrich (Steinheim, Germany). Methanol and acetonitrile (LiChrosolv®, gradient grade), ethanol and isopropanol (Emsure®), dimethyl sulfoxide (DMSO), Trifluoroacetic acid (TFA) and acrylamide were purchased from Merck KGaA (Darmstadt, Germany). HEPES buffer was from Invitrogen Co. (Scotland, UK).

*Encapsulation of P5 into liposomes*

P5 was encapsulated in liposomes following three different methods based on film-hydration procedure. For all formulations, lipids were used at a molar ratio of 15:2:3 (DMPC, DMPG, Chol) respectively and liposomes were prepared to contain 200 μg/mL P5 peptide and 40 mM of total lipid.

*Method A*

Lipids were dissolved in chloroform while P5 was dissolved in DMSO (10 μg/μL) and then they were combined in glass tubes and dried to a thin film by rotary evaporation (Heidolph, Germany) under reduced pressure. The lipid film was freeze-dried (VD-800F; Taitech, Japan) overnight to remove the solvents completely. The lipid film was then hydrated in HEPES buffer (10 mM, pH 7.2) containing 5% dextrose by intermittent vortexing and bath sonication under argon for a short time (approximately 30 sec at 25 ºC ) to disperse completely the lipids into the buffer. The resulting multilamellar vesicles (MLVs) were extruded 5 times through 400 nm and 11 times through 100 nm polycarbonate membranes at 25 ºC using a mini extruder (Avestin, Canada) to form 100 nm small unilamellar vesicles (SUVs) with a uniform size. To remove free peptide, 1 mL of liposomal dispersion was filled into the dialysis membrane (Cut off = 12-14 kDa) and dialyzed for 24 h at 4 ºC against 3 L of HEPES-dextrose buffer to allow the free peptide diffuse out. Vesicle size, polydispersity index and zeta potential of liposomes were determined by dynamic light scattering (Malvern Instruments, Malvern, UK). Liposomes were stored at 4 ºC under argon.

*Method B*

All the steps were the same as method A except that the lipid film was hydrated in HEPES-dextrose buffer containing 10% (v/v) of DMSO and peptide P5.

*Method C*

Lipid film was prepared as described in method A. The lipid film was dissolved in 300 μL ethanol and 700 μL HEPES-dextrose buffer containing 10% (v/v) of DMSO. The resulting dispersion was sonicated for about 15 s and extruded as explained in method A. 20 μL of P5 solution in DMSO was slowly added to preformed liposomes while vortexing. Subsequently, the ethanolic mixture of liposome and P5 was incubated at 25 ºC for 1 h and then dialyzed as described above to remove unencapsulated peptides, ethanol and DMSO. The effects of operating parameters of this method such as lipid concentration (10, 20, 40 and 60 mM), ethanol concentration (0, 10, 20, 30, 40 and 50% v/v), incubation temperature (4, 15, 25 and 40 ºC) and incubation time (0.5, 1, 1.5 and 2 hrs) on encapsulation efficiency were investigated.

*RP-HPLC analysis*

The analysis of peptide content of liposomes and their encapsulation efficiency were conducted by injection of different samples into a KNAUER smart line HPLC (Berlin, Germany). The RP-HPLC was equipped with a Nucleosil C18, 5 μm, 150 × 4.6 mm, 100 Aº column (KENAUER) and an UV detector (KENAUER S2600) set at 220 nm. The mobile phases employed were A (water + 0.1% TFA) and B (acetonitrile + 0.1% TFA). Elution program was a gradient starting with 100% A and increasing to 30% B in 2 min, 60%B in 12 min and 90% B in 2 min. The flow rate was set to 1 mL/min. The peptide concentration in all samples was determined using a standard curve generated by known concentrations of peptide dissolved in isopropanol containing 0.1% TFA.

*Determination of peptide recovery from liposomes using different solvents*

All experiments were set up in glass tubes in triplicate. Each tube was contained 10 μL of peptide solution and 500 μL of empty liposome containing 30% (v/v) ethanol. The mixture was incubated for 1 h at 25 ºC and dialyzed (Cut off =1000 Da) for 24 h at 4 ºC against 1.5 L of HEPES-dextrose buffer to remove ethanol and DMSO. Five hundred μL of methanol, ethanol, or isopropanol or 500 and 1000 μL isopropanol containing 0.1% TFA was added to 500 μL liposomes mixed with peptide in each tube. The tubes were vortexed and incubated at 40 ºC for 10 min. Three injections of 20 μL from each tube were made to the RP-HPLC. The amount of peptide in each injection was determined using a standard curve. For each extraction media, the average of peptide amount in 9 injections was put in equation 1 to calculate peptide recovery. 

REC% = (P Recovered / P Added) × 100                      equation (1)

Where REC% is the percent recovery, P Recovered is the amount of peptide recovered from liposomes and P Added is the amount of peptide added to empty liposomes.

*SDS-PAGE analysis of Lip-P5*

The analytical SDS-PAGE was carried out to determine qualitatively whether Lip-P5 contains P5 after purification. The gel consisted of running gel (16% (w/v) acrylamide / 6M urea), stacking gel (4% (w/v) acrylamide) and spacer gel (10% (w/v) acrylamide). The gel thickness was 0.7 mm. The anode buffer was 0.1 M Tris, pH 8.9 and cathode buffer was 0.1 M Tris, 0.1 M Tricine, 0.1% SDS, pH 8.25. Electrophoresis was carried out with an initial voltage of 30 V, which was gradually increased to 300 V at the end of the run. After electrophoresis the gels were stained for peptide with silver (23).

*Determination of encapsulation efficiency*

All the measurements were performed in triplicate in microcentrifuge tubes. Liposome suspensions (50 μL) were added to the tubes. Based on the P5 peptide recovery experiments, the optimized volume of the best extraction medium was added to the liposomes. The tubes were then vortexed and incubated at 40 ºC in a water bath for 10 min. Samples (20 μL; n=3) were injected to RP-HPLC. The peptide content in the injected volumes was determined by comparison with a standard curve for the peptide. Encapsulation efficiency (EE) was calculated using equation 2. 

EE% = (PLLiposome / PL Total) × (1/REC%) × 100                     equation (2)

Where EE% is the encapsulation efficiency of the peptide in liposomes, PL liposome is the peptide to phospholipids ratio (w/w) in liposomes and PL Total is the peptide to phospholipids ratio (w/w) used for formulation.

The amount of phospholipids was determined by using a phosphorus analysis spectrophotometric method (24, 25). Briefly, liposome samples (μL) containing theoretically 80 ± 50 nmol of phosphorus were placed into glass tubes. To each tube, 400 μL of a 10 N sulfuric acid solution was added and heated in an aluminum block at 200 ºC for 1 h. After cooling, 100 μL of a 0.1 mg/mL hydrogen peroxide solution was added and heated again for 10 min. The tubes were cooled and 500 μL of a 0.1 mg/mL ascorbic acid solution and 470 μL of a 2.2 mg/mL ammonium molibdate solution added to them. After vortexing, the solution was heated at 100 ºC in bath water for 10-20 min. The analyses were performed by reading the absorbance at 800 nm (Spekol 1500, analytikjena, Germany).

## Results

*Peptide recovery*

Peptide recovery from liposomes was determined using different solvents and applied as a criterion to select an efficient extraction medium. The highest peptide recovery value would indicate the most appropriate solvent to recover peptide from liposomes. For this purpose, a known amount of peptide was added to empty liposomes and extracted using methanol, ethanol, isopropanol or acidified isopropanol (Table 1).

**Table 1:** Peptide recovery from liposomes (n = 3; Mean ± SD).

**Recovery % (Mean ± SD) **	**Sample:Solvent ratio (v/v) **	**Solvent**
38.6 ± 3.9	1:1	Methanol
56.5 ± 3.6	1:1	Ethanol
67.2 ± 3.8	1:1	Isopropanol
80.3 ± 2.8	1:1	Isopropanol+0.1%TFA
92.6 ± 2.1	1:2	Isopropanol+0.1%TFA

Peptide was recovered from liposomes more efficiently using more hydrophobic solvents. P5 peptide recovery was the highest when isopropanol was used and followed by recovery with ethanol and then methanol. Surprisingly, peptide recovery with isopropanol was enhanced to 80% by adding 0.1%TFA. Extraction efficiency of this solvent for peptide recovery was also dependent on the volume ratio of sample to solvent. Acidified isopropanol with a sample to solvent ratio of 1:2 (v/v) was the most effective solvent system to recover P5 from liposomes.

*Liposome size, polydispersity index, zeta potential and encapsulation efficiency*

P5 peptide was encapsulated into liposomes using three different methods. For each method, liposome size, polydispersity index (pdI), zeta potential and encapsulation efficiency (EE) were determined as shown in Table 2.

**Table 2:** liposome size, pdI, zeta potential and encapsulation efficiency of peptide in liposomes (n = 3; Mean ± SD).

**Preparation Method**	**Liposome size (nm)**	**pdI**	**Zeta potential (mV)**	**Encapsulation efficiency (%)**
A	130.4 ± 8.7	0.156 ± 0.033	-42.3 ± 4.2	8.8 ± 3.4
B	118.8 ± 6.5	0.122 ± 0.028	-45.6 ± 3.8	17.5 ± 3.2
C	133.9 ± 11.8	0.142 ± 0.021	-40.8 ± 3.1	43.8 ± 1.9

All three methods led to forming liposomes with a size range of 110-150 nm which were desirable for vaccine formulations (26). Liposomes prepared using all the methods were also homogenous and had a uniform size with monomodal distribution (pdI < 0.2). However, the efficiency of different preparation methods to encapsulate P5 in liposomes was varied. The lowest encapsulation efficiency (8.8%) was seen by method A in which P5 was added directly to the lipid film. Efficiency of method B for encapsulating P5 in liposomes was only 14.5% .The highest encapsulation efficiency (44%) was obtained using method C in which P5 was added to the preformed liposomes in ethanolic dispersion.

*Confirmation of P5 encapsulation in liposomes by SDS-PAGE*

**Figure 1:**
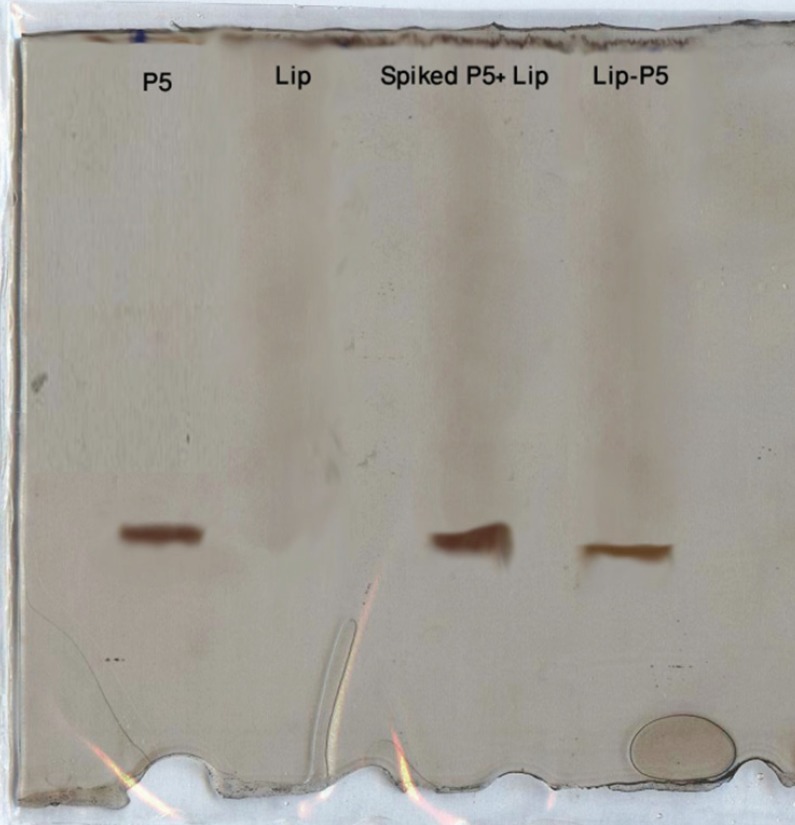
SDS-PAGE analysis of P5 peptide, empty liposome and liposome spiked with P5 and purified Lip-P5

The SDS-PAGE analysis of Lip-P5 prepared by method C after removing free peptide by dialysis revealed peptide encapsulation into the liposomes (Figure 1).

All three methods led to forming liposomes with a size range of 110-150 nm which were desirable for vaccine formulations (26). Liposomes prepared using all the methods were also homogenous and had a uniform size with monomodal distribution (pdI < 0.2). However, the efficiency of different preparation methods to encapsulate P5 in liposomes was varied. The lowest encapsulation efficiency (8.8%) was seen by method A in which P5 was added directly to the lipid film. Efficiency of method B for encapsulating P5 in liposomes was only 14.5% .The highest encapsulation efficiency (44%) was obtained using method C in which P5 was added to the preformed liposomes in ethanolic dispersion.

*Confirmation of P5 encapsulation in liposomes by SDS-PAGE*

The SDS-PAGE analysis of Lip-P5 prepared by method C after removing free peptide by dialysis revealed peptide encapsulation into the liposomes (Figure 1).

*Effects of lipid concentration, ethanol concentration, incubation time and incubation temperature on encapsulation efficiency*

**Figure 2:**
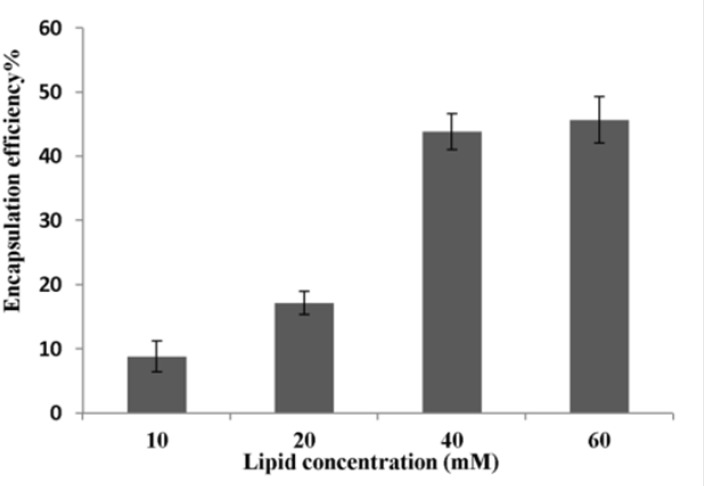
Effect of lipid concentration on P5 encapsulation efficiency (n = 3). P5 was encapsulated into liposomes using method C. Ethanol was used at concentration of 30% (v/v).The incubation temperature and incubation time were 25 ºC and 1 h, respectively

Figure 2 shows the effect of lipid concentration on P5 encapsulation efficiency obtained using method C. The ethanol concentration was fixed at 30% (v/v) and liposomes were incubated with peptide at 25 ºC for 1 h. The encapsulation efficiency increased from ca. 9% at 10 mM to 17% at 20 mM and finally to 44% at 40 mM lipid and plateaued at above 40 mM lipid.

**Figure 3:**
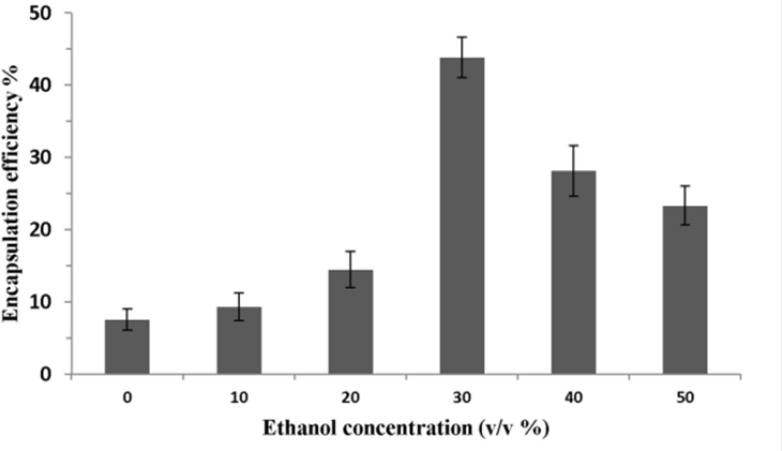
Effect of ethanol concentration on P5 encapsulation efficiency. The total lipid concentration was 40 mM and the ethanolic mixture of liposome and peptide was incubated at 25 ºC for 1 h. Error bars indicate the standard deviation of triplicate measurements

To determine the effect of ethanol concentration on encapsulation efficiency, P5 was added to liposomes (total lipid: 40 mM) containing different amount of ethanol and incubated at 25 ºC for 1 h. As seen in Figure 3, encapsulation efficiency was low (7.5%) when ethanol concentration was zero. It increased to 14.5% at the ethanol concentration of 20% and became highest at the presence of 30% ethanol. Interestingly, encapsulation efficiency dropped significantly as the ethanol concentration increased from 30% to 50% (v/v).

**Figure 4:**
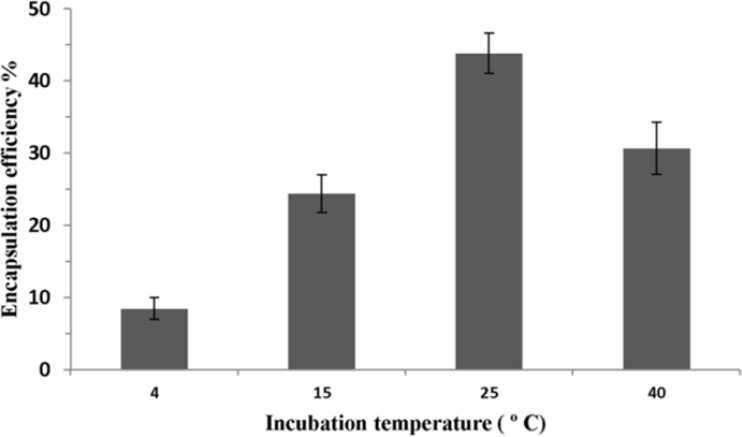
Effect of incubation temperature on P5 encapsulation efficiency. Incubation time was 1 h. Error bars indicate the standard deviation of triplicate measurements

Figure 4 shows the effect of incubation temperature on P5 encapsulation efficiency. Lipid concentration and ethanol concentration were fixed at 40 mM and 30% (v/v), respectively. Incubation time was also set for 1 h. The highest encapsulation efficiency was seen when the incubation temperature increased to 25 ºC. However, when the incubation temperature was increased to higher than 25 ºC, the encapsulation efficiency was decreased.

**Figure 5:**
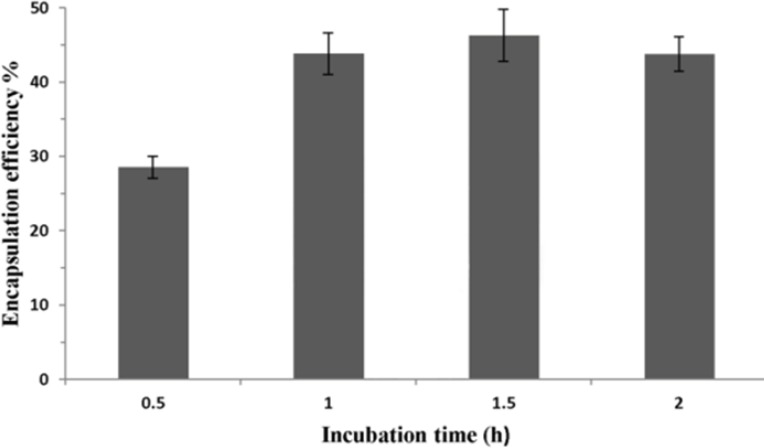
Effect of incubation time on P5 encapsulation efficiency. Lipid concentration and ethanol concentration were 40 mM and 30% (v/v), respectively. Incubation time was 1 h. Error bars indicate the standard deviation of triplicate measurements

To analyze the effect of incubation time on P5 encapsulation, 40 mM liposome containing 30% of ethanol and peptide were incubated at 25 ºC for different time. As shown in Figure 5, the longer incubation time increases the encapsulation efficiency of the peptide. However, if the incubation time is longer than 1 h, the amount of encapsulated peptide will reach to a plateau.

## Discussion

The ability of liposomes to enhance immune responses against encapsulated peptide and protein antigens has created a growing interest in developing peptide-based liposomal vaccines (27, 28). As the number of peptide-based vaccines under investigation escalates the development of methods to encapsulate peptide antigens into liposomes and characterize peptide formulations become more crucial.

In this study, three different procedures based on film-hydration method were used for encapsulating P5 antigen into liposomes. The results showed that that classical method of film hydration (method A) was not able to encapsulate P5 peptide effectively (Table 1). P5 is a small hydrophobic peptide and one might think P5 peptide would locate in the bilayer of liposomes. However, this is not the case for P5 peptide which is simple peptide without secondary structure. The polypeptide chain always crosses the lipid bilayer in a secondary structure like alpha-helical conformation or as beta barrel structure and not as a simple peptide strand, due to the presence of polarity in the peptide group (29). The other reason for the low encapsulation efficiency with method A could be due to the lack electrostatic interaction between P5 peptide and liposome surface. As Table 1 shows the liposome formulation used in this study is completely negatively charged. Based on P5 peptide pI value (pI 6.2) at pH 7.2, P5 peptide is also negatively charged. Therefore, there would be no electrostatic interaction between peptide and liposome interfaces to increase the encapsulation efficiency (8, 30). The loss of peptide could also occur during the extrusion process because of the very low solubility of P5 peptide (31).

Dissolving the peptide in buffer containing 10% (v/v) of DMSO (method B) allowed us to encapsulate more peptide into liposomes in comparison with method A. This indicates that inner volume of liposomes could be remarkable for encapsulation of the peptides. DMSO as a co-solvent enhanced P5 solubility in buffer; therefore, the encapsulation efficiency is higher. 

In method C, P5 was added to preformed liposomes in the presence of ethanol and DMSO and incubated at 25 ºC for 1h. Encapsulation efficiency of peptide was increased to ca. 44% following the strategy of using preformed liposomes for encapsulating. The SDS-PAGE analysis confirmed the encapsulation of P5 peptide in liposomes after purification (Figure 1). The presence of ethanol and DMSO increase the P5 peptide solubility substantially and also these two solvents make the liposome bilayer very fluid. Therefore, during incubation time at 25 ºC the peptide can penetrate easily into the liposome bilayer and entrap inside the liposomes. Moreover, using this method there would be no loss of peptide during the extrusion step.

Investigation on the effects of procedure parameters of method C on encapsulation efficiency demonstrated that this method is an optimized procedure for encapsulating P5 peptide for our used lipids in the formulation (Figures 2-5).

Increasing lipid concentration certainly led to an increase in the number of liposomes present per milliliter and, therefore, to an increase in the total inner volume of liposomes (32). This might explain why encapsulation efficiencies were improved for high lipid concentrations (Figure 2). Nevertheless, a saturation of this effect was observed on the total lipid concentration of 40 mM. This might be explained by the handling difficulties of dispersion at very high lipid concentrations and by the possible impediment of the viscosity of the medium, which could hamper the free movement of P5 into the inner cavity of liposomes (12, 33).

Figure 3 showed that ethanol concentration enhancement could have different effects on encapsulation efficiency of the peptide. Ethanol is a solvent of lipids and possess the surfactant property. It can enhance permeation of liposomal membranes and result in passive loading of P5 in the inner cavity of liposomes. The positive effect of ethanol concentration on encapsulation efficiency was observed when it was increased to 30% (v/v). However, if the concentration of ethanol is too high (> 30%), the encapsulation efficiency will became lower, because the high permeability of the bilayer would increase leakage of loaded peptide especially during dialysis procedure (34, 35).

As shown in Figure 4, encapsulation efficiency of peptide increased when the incubation temperature was raised to 25 ºC. This result can be explained by the fact that at higher temperatures, the structure of the lipid membrane could become more irregular and looser and peptide would pass through the bilayer easily. The maximal encapsulation efficiency of P5 was achieved at 25 ºC which is near the lipid phase transition temperature (Tm) of our main lipids, DMPC and DMPG, in the formulation where the liposomal membrane could become most permeable due to transient bilayer defects caused by the dramatic lateral area (and volume) changes produced by lipid domains as they fluctuate between the gel and fluid states in dynamic equilibrium (36). At temperature above Tm, the encapsulation efficiency dropped significantly. The bilayer structural rearrangements and fluctuation occurring in the thermotropic lipid chain melting transition are most likely responsible for the unusual temperature dependent kinetics of passive bilayer permeability (37). Meanwhile, the highest permeability of liposomal bilayers is in their Tm (38).

Encapsulation efficiency of peptide in liposomes showed to be time-dependent, when incubation time was increased to 1 h (Figure 5). Passive diffusion of peptide into permeable liposomes as loading mechanism can describe this dependency. The longer time peptide and liposomes were incubated, the more peptide diffused into liposomes. However, the amount of encapsulated peptide reached a plateau when the incubation time was longer than 1 h. Limited inner volume of liposomes or dynamic equilibrium achieved between free and loaded peptide over 1 h may contribute to reach this plateau.

In this study, we also examined the ability of several solvents to recover peptide from liposomes (Table 1). The results demonstrated that the amount of peptide recovered depended on solvent hydrophobicity. As P5 is a hydrophobic peptide, the more hydrophobic solvent we used, the more peptide was recovered (17). Peptide solubility in extraction medium may in fact influence peptide recovery from lipids. Adding TFA to isopropanol increased peptide recovery because TFA can increase peptide solubility in extraction medium. Furthermore, as more acidified isopropanol was used, more peptide could be solubilized. For P5, acidified isopropanol used at 1:2 of sample to solvent ratio (v/v) was the most valuable solvent. It recovered a maximal amount of peptide with a minimal amount of variation.

In conclusion, a simple up-scalable method was devised for the encapsulation of P5 peptide in liposomes. The methods of assay and encapsulation efficiency determination of P5 in liposomes were also optimized. P5 peptide liposomes could be helpful in developing liposomal vaccine formulations in terms of antitumor therapies in cancers in which HER2/neu antigen overexpresses.
